# EphA2 enhances the proliferation and invasion ability of LNCaP prostate cancer cells

**DOI:** 10.3892/ol.2014.2093

**Published:** 2014-04-25

**Authors:** PEIJIE CHEN, YAN HUANG, BO ZHANG, QIUQUAN WANG, PEIMING BAI

**Affiliations:** 1 Department of Urology, Zhongshan Hospital, Xiamen University, Xiamen, Fujian 361004, P.R. China; 2 Department of Chemistry, College of Chemistry and Chemical Engineering, Xiamen University, Xiamen, Fujian 361005, P.R. China

**Keywords:** prostate cancer, LNCaP cell, EphA2, cell proliferation, tumor invasion

## Abstract

EphA2 is persistently overexpressed and functionally changed in numerous human cancers. However, to the best of our knowledge, the role that EphA2 plays in prostate cancer is not entirely clear. To investigate the roles of EphA2 in the development and progression of prostate cancer, the present study initially evaluated the roles of the EphA2 protein in LNCaP prostate cancer cells using recombinant plasmid, western blot analysis, flow cytometry, Matrigel invasion chamber and the cell counting kit-8 assay. An immunohistochemistry assay was also conducted to observe the effects of EphA2 in prostate cancer tissues. The results demonstrated that the LNCaP human prostate cancer cells that were transfected with pcDNA3.1(+) plasmid-mediated pcDNA3.1(+)-EphA2, markedly enhanced the cell growth and invasion *in vitro*. Additionally, EphA2 was overexpressed in prostate cancer specimens and the expression of EphA2 was significantly associated with Gleason grade, total prostate-specific antigen, advanced clinical stage and lymph node metastasis. Collectively, these results demonstrate that EphA2 is involved in malignant cell behavior and is a potential therapeutic target in human prostate cancer.

## Introduction

EphA2, first reported by Lindberg and Hunter in 1990 (1), was obtained from the cDNA library screening of human keratin epithelial (HeLa cells) via a degenerate primer. The human EphA2 gene is located at chromosome 1p36.1 and is composed of 17 exons (2). EphA2 protein is a tyrosine kinase-containing transmembrane glycoprotein receptor with a molecular weight of 130 kDa and it belongs to the Eph receptor tyrosine kinases family. EphA2 is extensively expressed in the epithelial and endothelial cells of normal vessels (3) with a low abundance in the phosphorylated form (1). Compared with normal tissues, the EphA2 receptor tyrosine kinase is expressed relatively higher in the tissues of the prostate, breast, endometrial, gliofibroma, ovarian, bladder and renal carcinomas in the non-phosphorylated form (4–10). In these cancers, high levels of EphA2 predict metastasis and a poor survival rate (4–10). It was reported that the therapeutic delivery of EphA2 siRNA into an orthotopic mouse model of ovarian cancer diminished tumor growth in comparison to non-silencing siRNA (8). The strong correlation between the critical factors involved in angiogenesis and invasion, including a greater microvessel density and higher matrix metalloproteinase, with an enhanced expression of EphA2 was also revealed in ovarian cancer samples (11). Additionally, the positive role of EphA2 was demonstrated during mammary tumor onset and growth in the MMTV-Neu transgenic mice model, but not in mice overexpressing the polyomavirus middle T antigen. This indicates that, at least in breast carcinoma, the role of EphA2 in tumor progression is determined by the oncogene/tumor suppressor context (12). Previously, Taddei *et al* (13) revealed that EphA2 in prostate carcinoma cells activates the metastatic growth that regulates amoeboid motility and clonogenic potential.

The aforementioned studies have supplied data that increases the understanding of the role of EphA2 in malignant tumor behavior. The aim of the present study was to introduce EphA2 into a non-expressing prostate cancer cell line to investigate the direct potential of EphA2 on promoting malignant behavior. It has been previously demonstrated that the expression of EphA2 was at undetectable levels in LNCaP human prostate cancer cells and was highly overexpressed in PC-3 prostate cancer cells by western blot analysis (7). To understand this further, the exogenous EphA2 gene-transfected LNCaP cells were used to generate an EphA2 overexpressed cell line, and the effect of EphA2 overexpression on proliferation and invasion ability was investigated in the LNCaP prostate cancer cells. An immunohistochemical assay was also conducted to observe the effects of EphA2 in prostate cancer tissues. Taken together, these data indicate that EphA2 may be a novel and significant target for clinical intervention against prostate cancer.

## Materials and methods

### Recombinant plasmid pcDNA3.1(+)-EphA2 identification and amplification

The Agarose Gel DNA Purification kit, version 2.0 (Takara Biotechnology Co., Ltd., Dalian, Liaoning, China) was used to purify the *Hin*dIII/*Xho*I double-digested pcDNA3.1(+) plasmid (Invitrogen Life Technologies, Carlsbad, CA, USA). The purified linear pcDNA3.1(+) plasmid and EphA2 cDNA (Invitrogen Life Technologies) were linked using the DNA Ligation kit, version 2.0 (Takara Biotechnology Co., Ltd.). Following the determination of the plasmid purity with UV spectrophotometry (DU-800 spectrophotometer; Beckman Coulter, Fullerton, CA, USA), the sample was sent to the Shanghai Gene Co., Ltd., (Shanghai, China) for identification. According to the manufacturer’s instructions for the endotoxic plasmid extraction kit (Tiangen Biotech Co., Ltd., Beijing, China), the positive clone was extracted. The plasmid purity and concentration of the DNA product was determined by spectrophotometry.

### Cell culture and generation of EphA2-expressing stable LNCaP cells

The human prostate cancer cell lines, LNCaP and PC-3, were obtained from the Shanghai Cell Culture Collection (Shanghai, China). The zero-loaded plasmid, pcDNA3.1(+), and recombinant plasmid, pcDNA3.1(+)-EphA2, were transfected into LNCaP cells, respectively, using the Lipofectamine 2000™ transfection reagent (Invitrogen Life Technologies) according to the manufacturer’s instructions. Furthermore, the transfected cells were selected by adding G418 (500 μg/ml; Invitrogen Life Technologies), while the EphA2 expression level was confirmed by western blot analysis. Monoclones were subcultured and were labeled as LNCaP (blank), LNCaP-pcDNA3.1(+) (negative) and LNCaP-EphA2 (test), respectively. The cells were maintained in RPMI-1640 (Invitrogen Life Technologies) supplemented with 10% fetal bovine serum and 1% penicillin/streptomycin (both Invitrogen Life Technologies).

### Determination of the effect of EphA2 on the proliferation and invasion activity of the LNCaP cells

Following 24 h of serum-free starvation of the three cell lines in the exponential phase, the matrix was added. Following incubation for 48 h, the cells were collected. Subsequently, the samples were washed with PBS at an ambient temperature, immobilized with 75% ethanol and stored at −20°C prior to the removal of ethanol via centrifugation for 5 min at 1,000 × g. The samples were resuspended with PBS for 15 min at an ambient temperature and then centrifuged. Subsequently, 1 ml propidium iodide (MultiSciences (LIANKE) Biotech Co., Ltd., Hangzhou, China) was added, incubated for 30 min at an ambient temperature and flow cytometry was performed using BD FACSCanto II flow cytometer (BD Biosciences, San Jose CA, USA).

According to the manufacturer’s instructions for the Cell Counting Kit-8 (CCK8; Nanjing Kaiji Biology Development Co., Ltd., Nanjing, China), the three LNCaP cell lines were collected at the exponential phase and inoculated onto a 96-well plate at a density of 3,000 cells/well, with each cell line in six wells. Following inoculation, each well was replaced with 100 μl RPMI-1640 supplemented with 10% fetal bovine serum and 1% penicillin/streptomycin, and 10 μl CCK8 for 1–5 days. Subsequently, the cells were incubated at 37°C for 3 h, followed by ELISA for A450 determination.

Invasion activities of the three cell lines were determined according to the BioCoat Matrigel Invasion Chamber guide (BD Biosciences). The cells were inoculated at a density of 5×10^4^ cells/well and incubated at 37°C for 24 h. The cells were fixed with formaldehyde (Shanghai Bogoo Biotech. Co., Ltd., Shanghai, China), stained and counted under a microscope (Olympus IX51; Olympus, Nagano, Japan) with five fields of vision. The invasion activity of the carcinoma cells was characterized with the average transmembrane cell numbers. Each experiment was repeated three times.

### Western blot analysis

Western blotting was performed as described previously (14). Equal amounts of protein (30 μg) were size-fractionated on 10% SDS-PAGE (Solarbio Biotech Co., Ltd., Beijing, China) and transferred to nitrocellulose membranes (Pierce Biotechnology, Inc., Rockford, IL, USA) overnight. Samples were incubated with EphA2 rat anti-human monoclonal antibody (1:500; Abcam, Cambridge, UK) and β-actin mouse anti-human monoclonal antibody (1:1,000; MultiSciences (LIANKE) Biotech Co., Ltd.) at 4°C overnight. Subsequently, horseradish peroxidase-labeled goat anti-rat (1:20,000; Abcam) and goat anti-mouse secondary polyclonal antibodies (1:3,000; MultiSciences (LIANKE) Biotech Co., Ltd.) were added respectively, followed by Immobilon enhanced chemiluminescence (Merck Millipore, Billerica, MA, USA).

### Tissue specimens

A total of 86 patients with prostate cancer and 40 benign prostate hyperplasia control subjects were recruited between January 2003 and December 2012 at the Zhongshan Hospital, Xiamen University (Xiamen, Fujian, China). None of the patients received any anticancer therapy prior to the sample collection. The median age of patients was 68 years (range, 54–79 years), and the median level of serum total prostate-specific antigen (tPSA) was 31.52 ng/ml (range, 5.35–95.7 ng/ml). The clinical and pathological characteristics are shown in Table I. Clinical stage is classified according to the Jwett-Whitmore-prout classification (15).

### Immunohistochemical analysis

The EphA2 status was found using the established immunohistochemistry method of the avidin-biotin-peroxidase complex assay, as described by Zeng *et al* (16). Immunohistochemical analysis of EphA2 expression in prostate carcinoma and benign prostate hyperplasia samples was performed with a monoclonal mouse anti-human-EphA2 antibody (1:100; Abnova Corporation, Taipei, Taiwan), followed by biotinylated goat anti-mouse immunoglobulin G (1:1,000; Maixin Biotechnology Development Co., Ltd., Fuzhou, China) and peroxidase-labeled streptavidin. The controls were obtained from the Department of Pathology of the Zhongshan Hospital (Xiamen University, Xiamen, China). The positive controls consisted of human breast cancer samples that have been shown to express EphA2 and LnCaP cells were used as negative controls. Semi-quantitative assessment of immunohistochemical expression was performed as previously described (17) by assessing the percentage of stained tumor cells and staining intensity. Briefly, the percentage of positively stained cells was rated as follows: 1 point, 0–10%; 2 points, 11–50%; 3 points, 51–75%; and 4 points, >75%. The staining intensity was set from 0–3 points (0, no staining; 1, weak staining; 2, moderate staining; and 3, strong staining). When the product of the scores for intensity and the percentage of positive cells was >3 points, EphA2 immunoreactivity was considered positive. For each sample, at least five fields were observed at a high power (x400) under an Olympus IX51 microscope (Olympus) to derive a score for EphA2 expression. The pathological and immunohistochemical outcomes were checked and approved by two pathologists independently in the Department of Pathology of the Zhongshan Hospital, Xiamen University.

### Statistical analysis

Data were processed with SPSS 13.0 software (SPSS, Inc., Chicago, IL, USA). Measurement variables were presented as the mean ± standard deviation and compared using Student’s t-test. Categorized variables were compared using the χ^2^ test, or Fisher’s exact test when the χ^2^ test was unavailable. P<0.05 was considered to indicate a statistically significant difference.

## Results

### EphA2 overexpression in LNCaP-EphA2 cells

**Figure 1:**
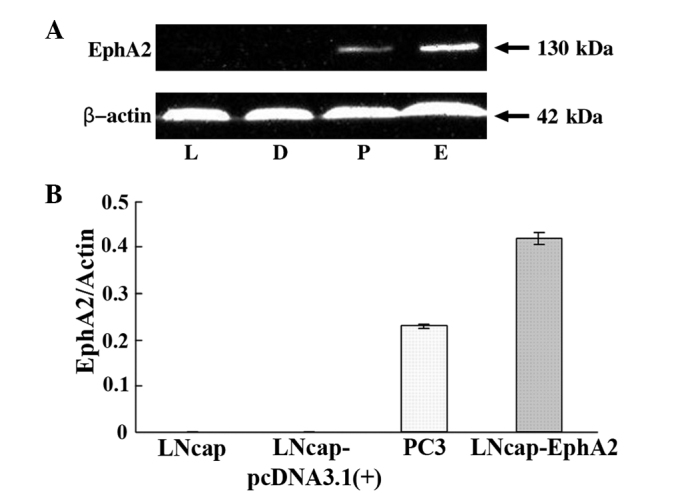
(A) EphA2 overexpression in LNCaP-EphA2 cells. Whole cell lysate was extracted and western blot analysis was performed to detect EphA2 expression. No band was shown at 130 kDa for LNCaP and LNCaP-pcDNA3.1(+) cell lines, but it was clearly visible for PC3 and LNCaP-EphA2 cell lines. (B) Ratios of EphA2 to actin in the four cell lines. The expression of EphA2 in LNCaP-EphA2 was stronger compared with that of PC3. L, LNCaP; D, LNCaP-pcDNA3.1(+); P, PC3; E, LNCaP-EphA2.

It has been previously demonstrated that EphA2 is expressed at high levels in the PC-3 prostate cancer cells compared with LNCaP cells (7). In the present study, whether the introduction of EphA2 into the LNCaP cells would promote proliferation and invasion behavior was investigated. Therefore, the recombinant plasmid, pcDNA3.1(+)-EphA2, or the zero-loaded plasmid, pcDNA3.1(+) control, was transfected by means of Lipofectamine 2000 transfection reagent into the LNCaP cells. Following G418 selection, EphA2 expression was confirmed by western blot analysis (Fig. 1A and B).

### EphA2 overexpression increases cell growth in vitro

**Figure 2:**
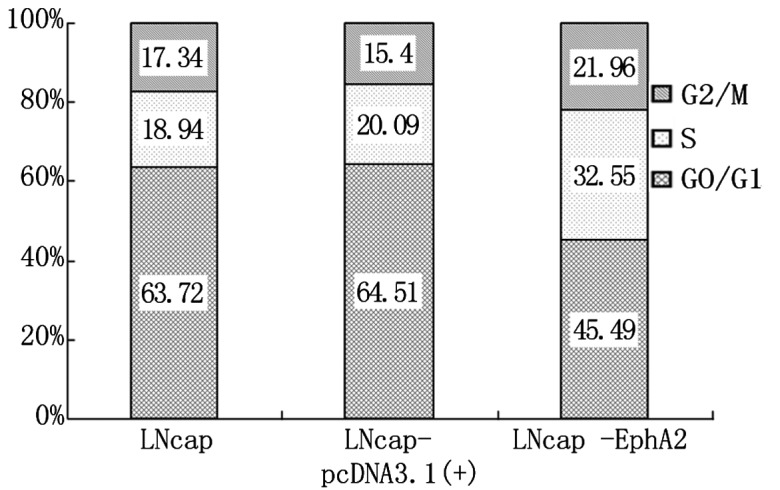
Cell phase distribution of various cell lines. The cell percentage at G0/G1 phase was significantly lower for the LNCaP-EphA2 than LNCaP and LNCaP-pcDNA3.1, and the cell percentage at phases S and G2/M was clearly increased, the difference was statistically significant (P<0.01). The cell percentage for LNCaP and LNCaP-pcDNA3.1(+) was similar at all phases, and its difference was statistically insignificant (P>0.05).

DNA cell cycle analysis was performed by cytometry for the three cell lines, LNCaP, LNCaP-pcDNA3.1(+) and LNCaP-EphA2. As shown in Fig. 2, the percentage of cells at the G0/G1 phase was significantly lower in LNCaP-EphA2 compared with LNCaP and LNCaP-pcDNA3.1 cells. The cell percentage at phases S and G2/M was clearly increased, and the difference was statistically significant (P<0.01). The cell percentage for LNCaP and LNCaP-pcDNA3.1(+) was similar at all phases, and its difference was statistically insignificant (P>0.05). The results show that EphA2 can enhance the proliferation of LNCaP cells.

### EphA2 overexpression promotes cell proliferation

**Figure 3:**
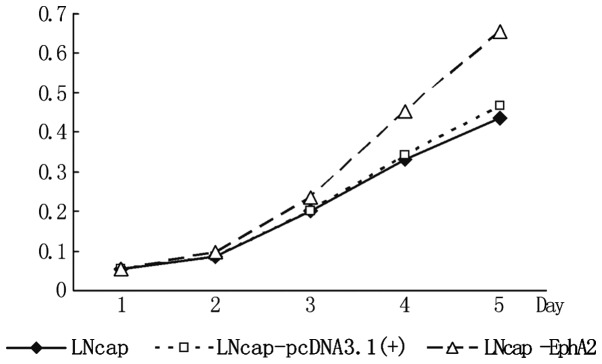
CCK8 test of cell proliferation. The CCK8 kit showed that the OD values of three cell lines showed no significant difference from day 1 to 2. From day 3, the OD of LNCaP-EphA2 cell line was significantly higher compared with the other two groups, and it became more clear on day 4 and 5; the difference was statistically significant (P<0.01). CCK8, Cell Counting Kit-8; OD, optical density.

As shown in Fig. 3, the optical density (OD) values of the three cell lines showed no significant difference from day 1 to 2. From day 3, the OD value of the LNCaP-EphA2 cell line was significantly higher compared with that of the other two groups. This became clearer on days 4 and 5 and the difference was statistically significant (P<0.01). This result shows that EphA2 can significantly enhance the proliferation of the LNCaP cells.

### EphA2 overexpression enhances the invasion ability of the prostate cancer LNCaP cells

**Figure 4:**
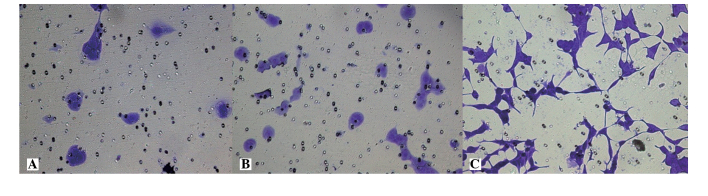
Transwell analysis of cell invasion activities. The invasion cell numbers of (C) LNCaP-EphA2 were significantly higher compared with the first two groups, (A) LNCaP and (B) LNCaP -pcDNA3.1(+), and the difference was statistically significant (P<0.01); magnification, ×200.

The membrane-penetrating cell numbers for the LNCaP, LNCaP-pcDNA3.1(+) and LNCaP-EphA2 cell lines were 20.42±4.35, 22.07±5.74 and 89.64±6.81 cells, respectively. The invasion activity difference of the first two groups was not statistically significant (P>0.05), while the invasion cell numbers of LNCaP-EphA2 was significantly higher compared with the first two groups and was statistically significant (P<0.01). The result shows that the cell invasion activity of EphA2-transfected LNCaP cells was significantly increased (Fig. 4).

### EphA2 overexpression is associated with aggressive features in patients with prostate cancer

**Figure 5:**
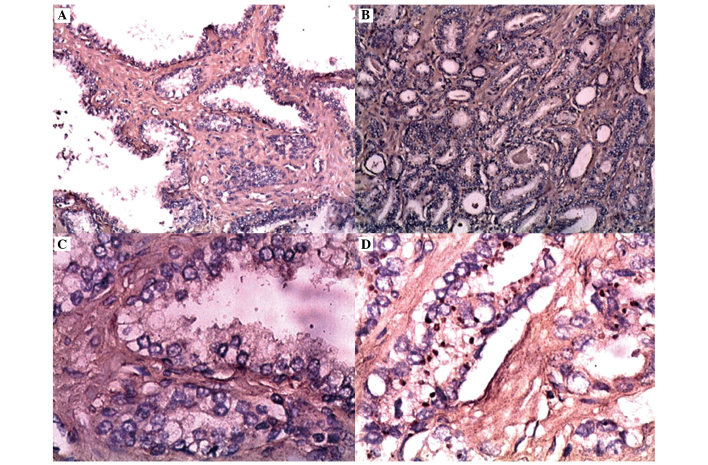
EphA2 immunohistochemistry in benign prostatic hyperplasia and prostate cancer. (A and C) The tissues of benign prostatic hyperplasia (magnification, ×100 and ×400, respectively). (B and D) The tissues of prostate cancer (magnification, ×100 and ×400, respectively).

Based on the findings that EphA2 enhances proliferation and invasion ability of the LNCaP prostate cancer cells, whether EphA2 overexpression is associated with aggressive features in patients with prostate cancer was assessed. Representative photomicrographs illustrating EphA2 expression in prostate cancer and benign prostate hyperplasia are presented in Fig. 5. The EphA2 immunoreactivity was distributed diffusely throughout the cytoplasm in the cells, with 12 prostate cancer samples observing both cytoplasmic and membrane staining. In the benign tissues, 10% of the samples demonstrated weakly positively stained cells (score of 1). The staining intensity of EphA2 was significantly higher (P<0.05) in carcinoma cells compared with that in benign tissues (Table II). In the prostate cancer cells, 72 (83.72%) samples had positive EphA2 immunoreactivity, with 65.74±4.51% as the mean of positively stained cells and 2.26±0.10 as the mean staining intensity.

Subsequent to finding high levels of EphA2 in prostate cancer, the level of EphA2 associated with known prognostic variables was assessed. The correlations between EphA2 overexpression and various clinical and pathological variables are listed in Table I. There was an increase in immunoreactivity with an increase in the stage and grade of the prostate carcinoma. The EphA2-positive immunoreactivity was significantly greater in C + D stages compared with A + B stages of the prostate cancer (P<0.05). Notably, high levels of EphA2 expression were also associated with higher-grade tumors (Gleason score ≥795.67%, P<0.05) and increasing level of tPSA (P<0.05). There was no significant difference in EphA2 expression among patients of various ages.

## Discussion

A major finding of the present study was that the plasmid delivery of the exogenous EphA2 gene (pcDNA3.1(+)-EphA2) in prostate cancer cells was sufficient to enhance LNCaP prostate cancer cell growth and invasion. In accordance with this data, Taddei *et al* (13) reported that EphA2 influences the metastatic growth that regulates amoeboid motility and the clonogenic potential in the prostate carcinoma cells. In fact, LNCaP-EphA2 enhanced the growth of the prostate cancer cells *in vitro* by >50% (Figs. 2 and 3). In a breast carcinoma study, Margaryan *et al* (18) reported that the expression of silence EphA2 can suppress carcinoma cell growth, invasion and vasculogenesis. This further indicates that EphA2 can be upregulated and that EphA2 may be a significant target in prostate cancer treatment. Parri *et al* (19) showed that EphA2 reexpression in melanoma cells converted their migration style from mesenchymal to amoeboid-like, conferring a plasticity in tumor cell invasiveness. Following reexpression and activation of EphA2, melanoma cells initiate a non-proteolytic invasive program that activates cytoskeleton motility, retracts cell protrusions, rounds the cell body and forces through the three-dimensional matrix to produce a successful lung and peritoneal lymph node metastases. The overexpression of EphA2 can enhance carcinoma cell invasion activity. This is realized through the weakening of intercellular connection and enhanced adhesion of carcinoma cells to the extracellular matrix and matrix invasion (20). This phenomenon may be relevant to the function of EphA2 in carcinoma cells. Although the EphA2 receptor is overexpressed, its distribution and phosphorylation are abnormal and it has a decreased affinity to its ligand. Therefore, it can not function properly. However, overexpressed EphA2 has a decreased sensitivity to its ligand, and so it can not negatively regulate the carcinoma growth and transport but it can enhance carcinoma cell migration and metastasis (21,22). To the best of our knowledge, this is the first *in vitro* study to show that the overexpression of the receptor by using a vector that contained EphA2 significantly upregulated the proliferation and invasion behavior in the LNCaP prostate cancer cell line.

Another novel outcome of the present study is that the overexpression of EphA2 in human prostate carcinoma cells is associated with aggressive features, including Gleason grade, tPSA and advanced clinical stage. Immunohistochemical analyses for EphA2 showed a much higher staining intensity in prostate carcinoma compared with normal tissues and a significantly increased expression with increasing stage of the disease and lymph node metastasis. The carcinoma pathological grade and invasion is correlated positively (23). Due to the overexpression of EphA2 in the majority of tumors and its suspected role in tumor growth and progression, EphA2 is being investigated as a novel therapeutic target. For example, EphA2 antibody treatment of athymic mice bearing MDA231 xenografts was sufficient to cause tumor regression with no adverse toxicity (24). Similar data in association with the growth inhibitory effects of EphA2 suppression have been illustrated in breast cancer, glioma and malignant mesothelioma (12,25,26), supporting the idea that EphA2 should be investigated as a target in other types of cancer, including prostate cancer.

In conclusion, the present study contributes more information on the function of EphA2 tumor growth and progression to further the understanding of the mechanism. The results of the present study show that the overexpression of EphA2 enhances the proliferation and invasion ability of the LNCaP prostate cancer cells. In addition, these results show that EphA2 overexpression is associated with aggressive features in patients with prostate cancer. The findings of the present study provide further support for developing novel therapeutic approaches targeted against EphA2 for patients with prostate cancer.

## Figures and Tables

**Table I:** Association between the expression of EphA2 and patient characteristics of prostate cancer.

Characteristics	n	Rate of positive EphA2 immunoreactivity, %	P-value
Age, years			
<60	36	83.33	>0.05
≥60	50	84.00	
tPSA, ng/ml			
<40	60	82.00	<0.05
≥40	26	96.24	
Lymph node metastasis			
Negative	64	76.17	<0.05
Positive	22	100.00	
Gleason grade			
<7	50	84.00	<0.05
≥7	36	95.67	
Clinical stage			
A + B	60	75.86	<0.05
C + D	26	100.00	

Total n=86. tPSA, total prostate-specific antigen.

**Table II:** Intensity of EphA2 antibody staining in the tissues of benign and malignant prostate cancer.

	EphA2 staining intensity			
	
Variable	0	1	2	3
Benign prostate hyperplasia, n=40	36	4	0	0
Prostate cancer, n=86	0	10	44	32

Staining intensity in prostate cancer was significantly higher compared with that in benign prostate hyperplasia (P<0.05).
